# Exosome-Derived miRNAs in Liquid Biopsy for Lung Cancer

**DOI:** 10.3390/life14121608

**Published:** 2024-12-04

**Authors:** Israel Martínez-Espinosa, José A. Serrato, Carlos Cabello-Gutiérrez, Ángeles Carlos-Reyes, Blanca Ortiz-Quintero

**Affiliations:** 1 Department of Molecular Biomedicine and Translational Research, Instituto Nacional de Enfermedades Respiratorias Ismael Cosío Villegas, Mexico City 14080, Mexico; 2 Department of Research in Virology and Mycology, Instituto Nacional de Enfermedades Respiratorias Ismael Cosío Villegas, Mexico City 14080, Mexico; 3 Laboratory of Onco-Immunobiology, Instituto Nacional de Enfermedades Respiratorias Ismael Cosío Villegas, Mexico City 14080, Mexico

**Keywords:** exosome-derived miRNAs, liquid biopsy, lung cancer

## Abstract

Exosome-derived microRNAs (miRNAs) are potential biomarkers for lung cancer detection and monitoring through liquid biopsy. These small, non-coding RNA molecules are found within exosomes, which are extracellular vesicles released from cells. Their stability in biofluids, such as blood, positions them as candidates for minimally invasive diagnostics. Multiple studies have shown that lung cancer patients exhibit distinct miRNA profiles compared to healthy individuals. This finding suggests that exosome-derived miRNAs could serve as valuable biomarkers for diagnosis, prognosis, and evaluating therapeutic responses. This review summarizes recent research on exosome-derived miRNAs in liquid biopsies, including blood, pleural effusion, and pleural lavage, as biomarkers for lung cancer, focusing on publications from the last five years.

## 1. Introduction

Lung cancer is currently the leading cause of cancer-related deaths worldwide and is often diagnosed late in its clinical course [1]. This delay significantly lowers the survival rate, as more advanced cases are less responsive to treatment [2,3]. Despite the availability of new drug combinations, multidisciplinary approaches, and advancements in precision medicine such as immunotherapy and targeted therapy, late-stage diagnoses and low survival rates remain challenging [4,5,6]. This underscores the need to identify specific biomarkers for detecting and monitoring lung cancer progression.

Tissue biopsies are crucial for confirming lung cancer diagnoses and identifying associated mutations and biomarkers. However, this procedure is invasive, unsuitable for repeated monitoring, and poses patient risks. Liquid biopsy has emerged as a potential and minimally invasive method for detecting and monitoring lung cancer [7,8]. Unlike traditional biopsies that require tissue samples, liquid biopsy involves identifying cancer-related biomarkers in body fluids, such as blood [9,10]. Liquid biopsies provide convenient access to samples and enable ongoing monitoring of disease progression over time. This is particularly important for tumors such as lung cancer, where tissue lesions may be in hard-to-reach areas. However, liquid biopsies come with certain limitations. They generally yield a lower quantity of biomarkers compared to solid tissue, and it is not possible to directly analyze the expression of genes, mRNA, and proteins from the tumor lesions themselves. On the other hand, components of liquid biopsies, such as circulating tumor DNA (ctDNA), circulating tumor cells (CTCs), and exosomes derived from tumor cells, can be used for mutation detection and for transcriptomic and proteomic analyses of the tumor landscape. However, challenges remain in standardizing sample collection procedures and the isolation of components to enhance yield, which is essential for effective clinical detection [9,10,11,12].

A key component of liquid biopsy involves exosome-derived microRNAs (miRNAs), which are consistently altered in patients with lung cancer compared to healthy individuals [13]. Recent research has identified these miRNAs as valuable markers for cancer detection due to their role in regulating genes associated with tumor development and progression [14,15]. Tumor cells selectively package these miRNAs into exosomes, which are then released into the bloodstream and other bodily fluids [15]. Exosomes can be isolated from such body fluids, and the miRNAs contained in these exosomes can be detected and quantified using highly sensitive molecular techniques [16]. Several studies have demonstrated the potential diagnostic value of these exosome-derived miRNAs, including their correlation with tumor stage, response to therapy, and overall patient survival [17,18,19].

This review summarizes recent research on miRNAs derived from exosomes in liquid biopsies, including blood, pleural effusion, and pleural lavage, as biomarkers for lung cancer, based on publications from the last five years. We established the following criteria for selecting studies: Inclusion Criteria: 1. Only peer-reviewed and indexed articles in PubMed published from 2020 to the present will be considered. 2. The studies must report experimental data on the biomarker value of exosome-derived miRNAs in body fluids, blood (serum/plasma), or liquid biopsies specifically for lung cancer. 3. Only articles with an established impact factor will be included. Exclusion Criteria: 1. Articles that are not peer-reviewed or indexed in PubMed. 2. Articles published before 2020. 3. Studies analyzing free extracellular miRNAs in body fluids. 4. Articles focused on other types of cancers. 5. Review articles.

## 2. Exosome-Derived miRNAs

### 2.1. Exosomes

**Figure 1:**
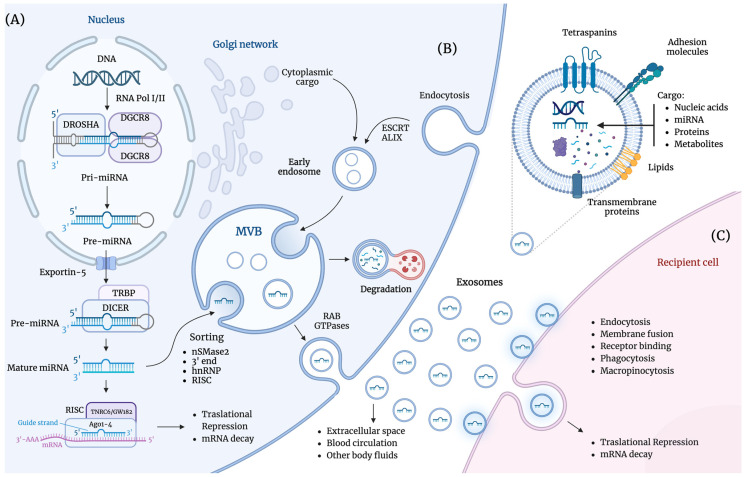
Illustration of (**A**) the canonical biogenesis of miRNAs, their processing, and the mechanisms of miRNA action; (**B**) the formation of exosomes, sorting of miRNAs into exosomes, and their release into the extracellular space; and (**C**) the uptake of exosomes by recipient cells, the transfer of miRNAs, and their function. A more detailed description is provided in the main body of the text. This figure was created using BioRender.com.

Exosomes are small vesicles bound by a lipid bilayer secreted by all types of cells. They carry various molecular contents, including proteins, lipids, DNAs, RNAs, and miRNAs. These vesicles measure 30–150 nm in diameter, mediate intercellular communication, and are crucial in maintaining various physiological and pathological processes [20,21]. Exosomes are formed when the cytoplasmic membrane buds inward to create early endosomes, which then develop into late endosomes (Figure 1B). These late endosomes contain several intraluminal vesicles (ILVs) that combine to form multivesicular bodies (MVBs). The endosomes incorporate lipids, soluble proteins, and membrane proteins from the extracellular environment during this process. The trans-Golgi network is also a source of the ILV contents and endocytic cargo [21,22]. The Endosomal Sorting Complex Required for Transport (ESCRT) and ALIX proteins are essential for cargo sorting and exosome formation. The ESCRT system is a conserved machinery composed of multiprotein complexes, including ESCRT-0, ESCRT-I, ESCRT-II, and ESCRT-III, along with accessory proteins such as VPS4, VTA1, and ALIX. These complexes function in a sequence to drive the formation of ILVs and assist in various processes that require membrane scission. ESCRT-0 is responsible for the initial recognition and sequestration of ubiquitinated cargo at the endosomal membrane. ESCRT-I acts as a structural and functional bridge between ESCRT-0 and ESCRT-II, organizing the clustering of cargo and facilitating membrane deformation. ESCRT-II further organizes cargo and recruits the ESCRT-III complex to carry out scission events. Finally, ESCRT-III forms spiral-shaped filaments that constrict the membrane and facilitate vesicle scission [23]. However, some studies indicate that exosome formation may occur independently of ESCRT, utilizing lipid-dependent pathways that involve tetraspanins, cholesterol, and ceramide [24]. The packaging of exosomes’ contents, including miRNAs, other non-coding RNAs, DNA, proteins, and lipids, is selective, indicating a process guided by specific molecular signals. Upon formation, MVBs can either be directed to lysosomes for degradation or transported to the plasma membrane to release their vesicles as exosomes into the extracellular space [24] (Figure 1B). Once in the extracellular space, recipient cells take up exosomes, transferring their contents. Recipient cells uptake exosomes through direct membrane fusion, endocytosis, micropinocytosis, phagocytosis, and receptor binding (Figure 1C) [15,25].

### 2.2. miRNAs

miRNAs are small, non-coding RNA molecules, typically 20–22 nucleotides long, that bind to complementary sequences on target mRNAs, resulting in either mRNA degradation or translational repression, modulating gene expression after transcription [26]. In canonical biogenesis, the transcription of miRNA genes by RNA polymerase II results in primary miRNA (pri-miRNA) [26,27] (Figure 1A). The pri-miRNAs undergo cleavage by the Drosha-DGCR8 complex within the nucleus, resulting in precursor miRNAs (pre-miRNAs). Pre-miRNAs are exported to the cytoplasm via exportin-5 and are processed by the RNase III enzyme Dicer to produce double-stranded mature miRNA [28,29]. The miRNA’s guide strand is incorporated into the RISC complex and binds to the target mRNA through partial sequence complementarity, leading to either degradation or translational inhibition of the target mRNA [30,31] (Figure 1A). Mature miRNAs are sorted into exosomes by mechanisms still under study. The proposed mechanisms include (a) the neural sphingomyelinase 2 (nSMase2)-dependent pathway; (b) the 3′ end miRNA sequence-dependent pathway; (c) the sumoylated heterogeneous nuclear ribonucleoproteins (hnRNPs)-dependent pathway; and (d) the RISC-related pathway [32,33,34]. The miRNAs carried by exosomes can modulate gene expression in the recipient cell by binding to complementary sequences in target mRNAs, leading to mRNA decay or the inhibition of translation (Figure 1C).

### 2.3. Role of Exosome-Derived miRNAs in Lung Cancer

**Figure 2:**
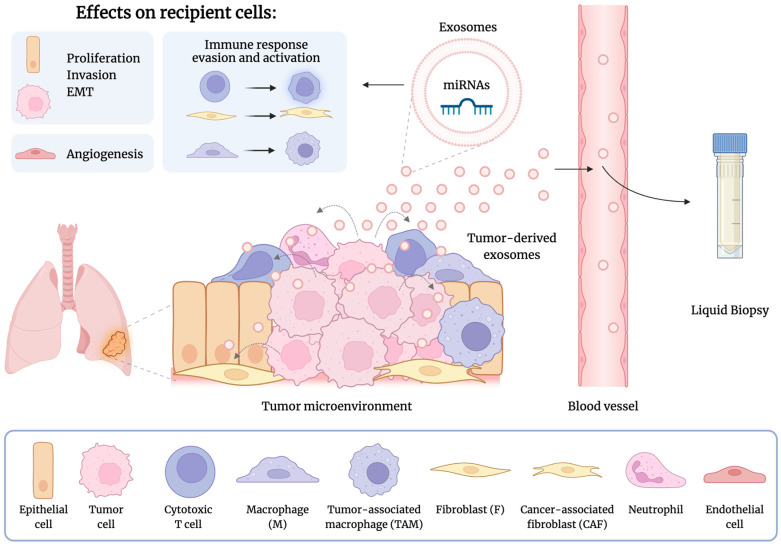
Illustration of the role of exosome-derived miRNAs in lung cancer. Lung cancer cells release exosomes containing miRNAs into the tumor microenvironment, bloodstream, and bodily fluids. These exosomes are taken up by surrounding recipient cells, including other tumor cells, normal lung epithelial cells, resident fibroblasts, immune cells, and nearby endothelial cells. Within the recipient cells, the exosome-derived miRNAs promote cancer-related processes such as cell proliferation, invasion, epithelial-to-mesenchymal transition (EMT), angiogenesis, cellular activation, and immune response evasion. Exosome-derived miRNAs are highly stable in body fluids, allowing them to be candidates for biomarkers in liquid biopsies. This figure was created using BioRender.com.

In cancer, tumor cells release exosomes enriched with miRNAs into the extracellular space, bloodstream, and other bodily fluids. In lung cancer, these exosome-derived miRNAs significantly impact various cancer-related processes in recipient cells. These processes include cellular proliferation, invasion, epithelial–mesenchymal transition (EMT), angiogenesis, immune evasion, and metastasis (see Figure 2) [35,36,37]. Moreover, exosome-derived miRNAs have been associated with regulating metastasis to distant organs, including brain metastasis in lung cancer [38]. Within the lung tumor microenvironment, tumor-derived exosomes are taken up by surrounding recipient cells, including other tumor cells, normal lung epithelial cells, resident fibroblasts, immune cells, and nearby endothelial cells (Figure 2). These exosomes can also travel through the bloodstream or other body fluids to reach target cells in remote locations, such as the brain and bones [35]. Significant populations within the tumor microenvironment, such as cancer-associated fibroblasts (CAFs) and tumor-associated macrophages (TAMs), also play a role in tumor regulation by secreting various factors, including exosome-derived miRNAs [35,39].

The biological effects of exosome-derived miRNAs released by lung tumor cells depend on the type of recipient cells and the specific target genes of the miRNAs. Research in lung cancer indicates that these miRNAs can promote cell proliferation, invasion, migration, and EMT in both lung tumor cells and normal epithelial lung cells. Furthermore, they help tumor cells evade immune responses by influencing resident behavior and infiltrating immune cells. They also play a role in angiogenesis by affecting vascular endothelial cells [35,40,41,42,43].

Therefore, exosome-derived miRNAs reflect the molecular changes associated with cancer development and progression, as they are involved in cancer-related biological processes and metastasis. These miRNAs are released by tumor cells into bodily fluids in a highly stable form. Their ability to indicate the molecular alterations occurring during cancer, their stability in biofluids, and their ease of sample collection make them promising candidates for minimally invasive biomarkers.

### 2.4. Exosome-Derived miRNAs in Liquid Biopsies for Lung Cancer

**Figure 3:**
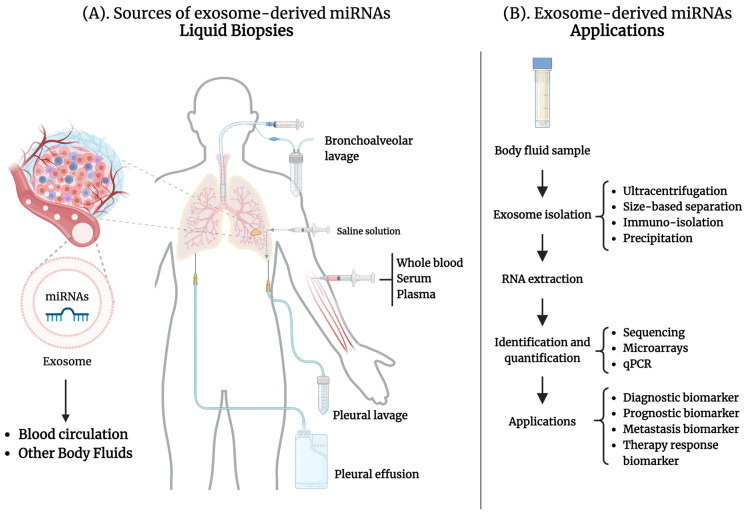
Exosome-derived miRNAs in Liquid Biopsies for Lung Cancer. Tumor cells release exosomes enriched with miRNAs to the blood circulation and other body fluids. A liquid biopsy involves analyzing bodily fluids to identify cancer-specific biomarkers, such as miRNAs derived from exosomes. In lung cancer, sources of exosome-derived miRNAs as biomarkers are whole blood, serum, plasma, pleural effusion, and bronchoalveolar and pleural lavages. Exosomes are isolated from body fluids using various methods, such as ultracentrifugation, size-based separation, or precipitation. After RNA extraction from exosomes, miRNAs can be identified and quantified by methods such as sequencing, microarrays, and qPCR. Specific exosome-derived miRNAs have demonstrated potential as biomarkers for diagnosis, prognosis, metastasis, and therapeutic response. The main body of the text provides a more detailed description. This figure was created using BioRender.com.

A liquid biopsy involves analyzing biofluids to identify cancer-specific biomarkers. The common sources of these biofluids include serum, plasma, saliva, urine, cerebrospinal fluid, stool, and pleural effusion [12,44]. In the case of lung cancer, exosome-derived miRNAs can be sourced from whole blood, plasma, serum, pleural effusion, pleural lavages, and bronchoalveolar lavage fluid (BALF) (see Figure 3A). However, the latest report on BALF was published in 2018.

The process begins with efficiently isolating exosomes from liquid biopsies and extracting RNA from those exosomes. Available techniques for exosome isolation include ultracentrifugation (the “gold standard”), size-exclusion chromatography, polymer precipitation, and immunoaffinity capture (Figure 3B). There are other emerging new technologies, such as microfluidic chips. These exosome isolation techniques present advantages and disadvantages, and the choice depends on the specific research objectives. A more detailed description of the currently available technologies for exosome isolation can be found here [45,46]. The next step is identifying and quantifying the miRNAs in the RNA sample with methods such as sequencing, microarrays, and quantitative polymerase chain reaction (qPCR). Finally, to analyze the potential biomarker value of these miRNAs, research should include a comparative analysis of samples from lung cancer patients and control subjects, with the characteristics of the controls and lung cancer patients tailored to align with the study’s objectives. Current studies indicate that exosome-derived miRNAs are potential biomarkers for diagnostic, prognostic, and predictive responses to therapy. Moreover, some miRNAs have demonstrated their value as biomarkers for monitoring tumor progression and predicting metastasis (Figure 3B).

The following sections describe the evidence from recent publications on the potential value of exosome-derived miRNAs found in liquid biopsies from lung cancer patients.

## 3. Exosome-Derived miRNAs from Peripheral Blood in Lung Cancer

### 3.1. Exosome-Derived miRNAs as Diagnostic and Prognostic Biomarkers

The latest literature presents various studies examining the diagnostic and prognostic value of exosome-derived miRNAs from the plasma of non-small-cell lung cancer (NSCLC) patients (Table 1). For instance, a study published in 2024 examined the diagnostic value of 25 exosome-derived miRNAs found in the plasma of patients with NSCLC. The research focused on a small cohort consisting of 36 patients with lung adenocarcinoma (AD), 36 patients with squamous-cell lung cancer (SCC), and 36 healthy controls. Using qPCR, the authors identified two unique signatures of exosome-derived miRNAs that effectively distinguish AD and SCC patients from healthy individuals. For adenocarcinoma, the combination of miR-126-3p, miR-221-3p, let-7b-5p, and miR-222-3p achieved an area under the curve (AUC) of 0.764. Meanwhile, the signature for SCC, comprising miR-21-5p, miR-221-3p, let-7b-5p, and miR-9-5p, yielded an AUC of 0.842. Overall, these combinations demonstrated better diagnostic performance than individual miRNAs [47]. However, the findings require validation in a larger cohort with additional patients and controls. Another study published in 2024 examined the potential of two miRNAs (miR-574-5p and miR-181a-5p), carried by plasma extracellular vesicles (EVs), to serve as prognostic biomarkers for patients with advanced NSCLC who are undergoing treatment with nivolumab, an immune checkpoint inhibitor targeting PD-1. The researchers utilized a microarray platform and qPCR to analyze the miRNA profiles derived from plasma EVs of 245 advanced NSCLC patients who received nivolumab as a second-line therapy. They developed a prognostic model that combined the EV-derived miRNAs with clinical variables. This model demonstrated that miR-181a-5p and miR-574-5p, along with the patient’s performance status, could effectively differentiate between patients unlikely to benefit from immunotherapy and those likely to benefit, predicting median overall survival (OS) times of 4 months or less versus those greater than 9 months, with an AUC of 0.76 [48]. These biomarkers may assist clinicians in predicting the effectiveness of nivolumab, thus helping to tailor therapy more effectively. In 2023, a study investigated the potential of two microRNAs, miR-1290 and miR-29c-3p, found in plasma exosomes as diagnostic markers for lung cancer (LC). RNA sequencing and qPCR were used to analyze a screening cohort of four LC and four benign lung disease (BLD) patients and a validation cohort of 30 NSCLC, eight small-cell lung cancer (SCLC), and 19 BLD patients. The results indicated that exosomal miR-1290 and miR-29c-3p effectively distinguish between LC and BLD, achieving areas under the curve (AUC) of 0.934 and 0.868, respectively. Furthermore, they differentiate between early-stage LC and BLD with AUCs of 0.947 and 0.895. They also distinguish NSCLC from SCLC, yielding a combined AUC of 0.860 [49]. In another study published in 2021, higher levels of plasma exosome-derived miR-1260b were associated with high-grade disease, metastasis, and poor survival of 48 NSCLC patients compared with 48 healthy controls. Kaplan–Meier survival analysis showed that patients with high exosomal miR-1260b levels had worse overall survival rates (*p* = 0.029) than those with low exosomal miR-1260b levels. A study published in 2021 found that elevated levels of plasma exosome-derived miR-1260b were linked to high-grade disease, metastasis, and poor survival outcomes in 48 patients with NSCLC compared to 48 healthy controls. Kaplan–Meier survival analysis indicated that patients with high levels of exosomal miR-1260b exhibited significantly worse overall survival rates (*p* = 0.029) than those with low levels of exosomal miR-1260b. However, this finding was not tested in a larger cohort [37]. In 2020, a study investigated the diagnostic potential of miR-342-5p and miR-574-5p, found in exosomes derived from the plasma of patients with early-stage AD, including pre- and post-operation patients, compared with healthy controls. Researchers observed elevated levels of miR-342-5p and miR-574-5p in early-stage AD patients (n = 56) compared to healthy controls (n = 40). The study reported a combined AUC of 0.813, with a sensitivity of 80.0% and a specificity of 73.2%. Additionally, the levels of these microRNAs significantly decreased after tumor resection [50]. Early-stage detection is critical for improving lung cancer outcomes, as early diagnosis can lead to more effective treatments. However, similarly to the previous study, these findings should be validated in a larger cohort.

The recent literature also includes studies exploring the diagnostic and prognostic value of exosome-derived miRNAs from the serum of LC patients (Table 1). For instance, a study from 2023 investigated a panel of exosome-derived miRNAs as potential diagnostic and prognostic biomarkers specifically for SCLC, a particularly aggressive type of lung cancer with a poor prognosis. A total of 126 SCLC patients and 50 healthy controls were analyzed by microarray and qPCR. The combination of miR-200b-3p, miR-3124-5p, and miR-92b-5p showed diagnostic value, with an AUC of 0.93. This three-miRNA panel was significantly associated with poor clinical outcomes (*p* = 0.0029) [19]. In 2023, another study analyzed the serum of 84 patients with NSCLC, 30 patients with benign lung lesions (BLL), and 47 healthy controls. Exosome-derived miRNA-4497 distinguished NSCLC patients from healthy controls with an AUC of 0.855 and NSCLC patients from BLL patients with an AUC of 0.748. Kaplan–Meier survival analysis indicated that overall survival (OS) was shorter in patients with low serum exosomal miR-4497 levels compared to those with high levels (low vs. high: 28.565 ± 3.815 months vs. 41.886 ± 2.759 months, *p* = 0.011) [51]. In 2020, a study found two diagnostic miRNAs in exosomes from the serum of 330 NSCLC patients compared with 312 healthy donors. Levels of miR-5684 and miR-125b-5p were downregulated in NSCLC, and the combined two miRNAs distinguished NSCLC patients from controls with an AUC of 0.793, a sensitivity of 82.7%, and a specificity of 62.1%. These combined two miRNAs also distinguished early-stage NSCLC from healthy controls with an AUC of 0.744, a relative sensitivity of 80.6%, and a relative specificity of 60.9%. The authors discovered that combining exosome-derived miRNAs with a carcinoembryonic antigen (CEA) and the cytokeratin 19 fragment (CYFRA21-1) enhanced the diagnostic performance of each marker to an AUC of 0.896 [52]. Another study published in 2020 reported that serum exosome-derived miR-7977 was upregulated in AD patients compared with healthy controls and distinguished them with an AUC of 0.787. The analysis was validated in a medium cohort of 62 AD patients and 62 healthy controls by qPCR [53]. In addition, a 2020 study analyzed the diagnostic and prognostic value of serum exosome-derived miR-378 in 103 patients with NSCLC and 60 control subjects. High levels of miR-378 were associated with a poorer prognosis, advanced disease, and worse survival rates. Furthermore, high levels of miR-378 differentiate NSCLC patients from controls with an AUC of 0.842, showing both diagnostic and prognostic value [54].

Most revised publications need validation in larger cohorts to establish their utility as biomarkers for future clinical applications. Among the miRNAs that show significant potential as lung cancer biomarkers in liquid biopsies are those from studies that have already included relatively large cohorts. For instance, prognostic plasma-derived exosomal miR-181a-5p and miR-574-5p were tested in 245 non-small-cell lung cancer (NSCLC) samples, while diagnostic serum-derived miR-5684 and miR-125b-5p were tested in 330 NSCLC samples and 332 control subjects. These studies would require further analytical and clinical validation on independent cohorts across multi-center sources. The appropriate metrics for evaluation will depend on the study’s objectives, but they should include consistent measurements relative to the true unknown values, interlaboratory consistency, and consideration of factors such as age, race, comorbidities, gender, and lifestyle across patients and controls. Additionally, diagnostic miR-3124-5p and miR-92b-5p were evaluated in 126 samples of SCLC. This is particularly significant considering the relatively low prevalence of SCLC among lung cancer cases. However, its relevance is heightened due to the aggressive nature of this type of cancer.

### 3.2. Exosome-Derived miRNAs as Metastasis Biomarkers

Currently, most potential miRNA biomarkers for lung cancer metastasis are found in tissues obtained through invasive procedures [35,36]. Exosome-derived miRNAs in liquid biopsies offer valuable opportunities to develop biomarkers for predicting lung cancer metastasis using minimally invasive methods. Monitoring these biomarkers could enhance the ability to identify at-risk patients and tailor more effective treatment strategies. For instance, serum exosome-derived miR-125b-5p demonstrated a diagnostic accuracy for metastatic versus non-metastatic NSCLC patients, with an AUC of 0.647 and sensitivity and specificity values of 50.6% and 70.4%, respectively. This 2020 study analyzed 330 NSCLC patients [52]. Another study published in 2020 showed that a combination of two plasma exosome-derived miRNAs (miR-320a and miR-622), with CEA and Cyfra21-1 markers, differentiate NSCLC patients with metastasis from those without metastasis with an AUC of 0.9. This study analyzed a medium cohort of 80 NSCLC patients [55].

Specific miRNAs found in exosomes are associated with the metastatic potential of lung cancer cells. Due to their involvement in metastasis, these miRNAs may serve as potential biomarkers. For instance, exosomal miR-1260b increases tumor metastasis by inhibiting the tumor suppressor HIPK2. It was found that high levels of miR-1260b could differentiate NSCLC patients with metastasis from those without metastasis (*p* < 0.05) in a cohort of 48 NSCLC patients [37].

While further research is required to validate these findings in larger patient populations, various stages of lung cancer, and through longitudinal studies, the potential benefits of exosome-derived miRNAs as biomarkers for metastasis are considerable. Additionally, one study showed that specific exosome-derived miRNAs can enhance the biomarker performance of established cancer markers, indicating a potential for combinatory biomarker effectiveness. For example, the combination of miR-320a and miR-622 with CEA and Cyfra21-1 was able to distinguish between metastasis and non-metastasis in NSCLC, achieving an area under the curve (AUC) of 0.9.

### 3.3. Exosome-Derived miRNAs as Therapeutic Response Biomarkers

Therapeutic resistance, especially to chemotherapy and targeted therapies, presents a significant challenge in managing lung cancer patients. The complexity of tumor dynamics and the heterogeneity of cancer cells contribute to this resistance, as some cells develop mechanisms to evade treatment effects. In recent years, analyzing exosome-derived miRNAs from liquid biopsies has emerged as a promising, minimally invasive method for assessing therapeutic response in lung cancer (Table 2). For example, in a 2021 study, researchers examined the association of exosome-derived miRNAs to resistance to osimertinib, a third-generation EGFR tyrosine kinase inhibitor (TKI), in NSCLC. The authors analyzed the miRNA profiles derived from exosomes of an osimertinib-resistant NSCLC cell line named H1975-OR compared to a drug-sensitive cell line designated H1975. They compared these findings with exosome-derived serum samples from 67 NSCLC patients who developed resistance to osimertinib during treatment. Elevated levels of miR-184 and miR-3913-5p in the exosomes of patients’ serum correlated with osimertinib resistance, particularly in those with EGFR exon 21 L858R- and T790M-positive mutations. Combining the two miRNAs resulted in an AUC of 0.7059 for resistance to osimertinib in patients with NSCLC. In NSCLC patients with mutations in L858R in the EGFR exon 21, exosome-derived miR-184 achieved an AUC of 0.736, while exosome-derived miR-3913-5p had an AUC of 0.759 [56]. A 2020 study examined miRNAs in plasma-derived EVs as potential biomarkers to predict responses to anti–PD-1/PD-L1 therapies in patients with advanced NSCLC. The study included 14 patients who responded (partial response or stable disease for at least six months) and 15 who did not (progressive disease), including a validation cohort of eight responders and 13 non-responders. A combination of decreased levels of miR-199a-3p, miR-21-5p, and miR-28-5p achieved an AUC of 0.925 to indicate a response to therapy [57]. A 2020 study examined plasma exosome-derived miRNAs as biomarkers to predict the response of advanced EGFR/ALK wild-type NSCLC patients to PD-1/PD-L1 immunotherapy. This study included 30 patients with EGFR/ALK wild-type NSCLC who received PD-1/PD-L1 inhibitors, and exosome-derived miRNAs were analyzed by next-generation sequencing. The results indicated that miR-320d, miR-320c, miR-320b, and miR-125b-5p were upregulated in non-responders, with a log CPM > 4 cut-off, *p* < 0.05, and FDR ≤ 0.1 [18]. However, these findings are based solely on sequencing data and have not undergone validation through a quantitative method such as qPCR. Additionally, a performance value was not analyzed. In a 2021 study, researchers used next-generation sequencing to analyze the profile of exosome-derived miRNA from the plasma of eight NSCLC patients when they became sensitive to osimertinib and after developing resistance. The results were validated in another cohort of 19 NSCLC patients by qPCR. They found upregulated levels of miR-323-3p, miR-1468-3p, miR-5189-5p, and miR-6513-5p in osimertinib-resistant NSCLC patients (*p* < 0.0001) [58].

While the results are promising, current studies highlight the necessity for larger cohorts and possibly multi-center trials to confirm the effectiveness and consistency of these biomarkers in lung cancer. Preliminarily, miR-199a-3p, miR-21–5p, and miR-28–5p may serve as promising predictive biomarkers for response to anti-PD-1/PD-L1 treatment, with a notable AUC of 0.925 indicating strong discriminatory performance.

## 4. Exosome-Derived miRNAs from Other Body Fluids as Biomarkers

Exosome-derived miRNAs are stable in various body fluids beyond just peripheral blood. Recent studies on lung cancer have focused on assessing their potential as biomarkers in pleural effusion and pleural lavage (see Table 3). Additionally, earlier research has examined these miRNAs in bronchoalveolar lavage [59,60].

A 2022 study examined the diagnostic potential of exosome-derived miRNAs in differentiating malignant pleural effusion due to lung adenocarcinoma (AD) from tuberculous pleural effusion. The study analyzed a small cohort of six AD and six tuberculosis (TB) patients. Results indicated that levels of miR-150-5p and miR-3614-5p were downregulated, while miR-200b-3p, miR-182-5p, and miR-629-5p were upregulated in AD compared with TB patients (*p* < 0.05) [61]. The findings suggested that these miRNAs may distinguish between AD and TB patients; however, the cohort size was too small. The results require validation in a larger, independent cohort. Additionally, the performance values of the biomarkers have yet to be evaluated. A 2020 study focuses on EV-derived miRNA-21 as a diagnostic factor for detecting pleural malignant dissemination of AD. Researchers examined the pleural lavage fluid of 41 AD patients using digital PCR. High levels of EV-miR-21 were linked to positive cytology and pleural invasion at primary sites, even in cases with negative cytology, suggesting its potential for early detection [62]. Another recent study investigated EV-derived miRNAs from the pleural lavage of 25 control patients with benign pleural effusions (BPE) and 21 patients with AD or SCC. Results indicated that miR-1-3p, miR-144-5p, and miR-150-5p levels can differentiate NSCLC from BPE patients with an AUC of 0.914, 0.925, and 0.939, respectively [63].

These recent studies emphasize the need for larger cohorts and the assessment of biomarker performance to confirm the diagnostic value of the identified miRNAs. Among them, miR-1-3p, miR-144-5p, and miR-150-5p demonstrated significant diagnostic value for NSCLC, showing a notable AUC of 0.9; however, this was based on a small study cohort.

## 5. Conclusions and Perspective

Exosome-derived miRNAs are increasingly recognized as valuable biomarkers for liquid biopsy applications in lung cancer. This type of cancer is often diagnosed at an advanced stage due to a lack of early symptoms and limited minimally invasive diagnostic tools. The high mortality rate associated with lung cancer is largely due to such late diagnoses, which leave few effective treatment options. Consequently, miRNAs obtained from exosomes in bodily fluids, such as blood, have emerged as a promising approach for minimally invasive diagnosis and disease monitoring.

As recently reviewed research revealed, exosome-derived miRNAs have potential use as biomarkers for early diagnosis, differential diagnosis, prognostic assessment, and monitoring of metastasis and therapeutic responses. This highlights the importance of integrating these miRNAs into clinical practice. However, the literature review indicates that while the field shows promise, it is still evolving concerning lung cancer. Most published studies rely on small- and medium-sized cohort analyses of exosome-derived miRNAs in liquid biopsies, and these findings often lack validation in larger, independent cohorts. Additionally, variability in miRNA profiles due to factors such as age, lifestyle, and comorbidities must be accounted for in studies to ensure an accurate interpretation of results. The analysis of the effects of different cancer stages, histological subtypes, mutations, risk factors, race, background, and gender could be significantly improved with larger cohorts. Expanding the size of these groups would enhance our understanding and provide more accurate insights into these critical factors. In addition, there is an opportunity to enhance our understanding of tumor dynamics, metastatic potential, recurrence, and the development of therapeutic resistance through longitudinal, large-scale studies. Conducting this research could significantly advance our knowledge and improve treatment outcomes.

In addition to the limitations posed by small study cohorts in recent lung cancer research, there are other significant challenges that need to be addressed. The validation and standardization of body fluid sampling processes, as well as the methods for exosome isolation, characterization, yield acquisition, and functional studies, are technically complex. The first comprehensive guidelines for standardization, known as the ‘Minimal Information for Studies of Extracellular Vesicles’ (MISEV), were published in 2014, with updated versions released in 2018 and 2023 [64]. The gold standard method for isolating exosomes is ultracentrifugation, which requires expensive equipment, is time-consuming (often taking hours), and necessitates large sample volumes to obtain a sufficient yield for subsequent miRNA purification, identification, and quantification. In addition, studies have shown that various methods are used for the initial identification of miRNAs isolated from exosomes. However, it is important to highlight that quantitative validation of miRNAs is consistently performed across these studies using qPCR.

It is important to note that significant evidence supports the potential usefulness of exosome-derived miRNAs as biomarkers in liquid biopsies. This evidence also suggests that these biomarkers may be more effectively implemented in clinical practice either as complementary tools alongside other markers, such as CEA, complementary to tissue biopsy, or as an alternative to tissue biopsy when the latter is not feasible, particularly for monitoring cancer progression or therapy response.

However, the question of whether miRNA-based liquid biopsy can serve as a sufficient method for the early diagnosis and confirmation of lung cancer can only be resolved through large, multi-center trials, which are currently lacking for lung cancer. If such trials were conducted, they could significantly advance lung cancer diagnostics, as late detection is the primary challenge associated with high mortality rates in this disease. While miRNA-based liquid biopsy holds great promise, confirming lung cancer diagnosis reliably requires a combination of clinical assessments, analytical techniques, and imaging methods. The implementation of liquid biopsy methods in clinical practice will only occur after extensive controlled studies are conducted, along with standardized techniques for sampling, exosome isolation, and miRNA quantification, which could become routine in clinics.

Our review aims to summarize recent research on miRNAs derived from exosomes in liquid biopsies, but it does have some limitations. Notably, we have excluded studies from previous years, which may result in missing valuable information. This decision was made because reliable advances in exosome isolation and characterization—the crucial first step in this field—have only emerged recently. Although the standardization of isolation methods and the characterization of exosomes are still in progress, only in the last few years have researchers begun using more reliable approaches for exosome nomenclature, isolation, characterization, and functional studies [64]. Another limitation of our review is the lack of discussion regarding the potential consequences of using different methods for isolating vesicles, the source of body fluids (such as serum vs. plasma), and the different approaches for identifying and quantifying miRNAs. Our review primarily focuses on the reported results of the studies and the potential performance of the biomarkers. To address these issues related to exosomes, we recommend consulting the latest advancements in the basic principles and advanced techniques for extracellular vesicle research, as outlined in “Minimal Information for Studies of Extracellular Vesicles (MISEV2023): From Basic to Advanced Approaches” [64].

In conclusion, the field of exosome-derived miRNAs in lung cancer liquid biopsy is promising but still developing. To enhance our understanding and application of these findings, it is essential for future research to focus on conducting large-scale studies and initiating further clinical trials. With these efforts, exosome-derived miRNAs may become key components in personalized and precision medicine approaches for lung cancer, contributing to improvements in early detection, monitoring, and patient prognosis.

## Figures and Tables

**Table 1:** Exosome-derived miRNAs from peripheral blood identified as potential diagnostic and prognostic biomarkers for lung cancer in the last five years of publications.

miRNAs	Sample	Cohort Size (n)	Application	Biomarker Performance Value	Ref
miR-126-3p, miR-221-5p, Let-7b-5p, and miR-222-3p	Plasma exosomes	AD (36), Ctr (36)	Diagnostic AD vs. Ctr	AUC = 0.764	[47]
miR-21-5p, miR-221-5p, Let-7b-, and miR-9-5p	Plasma exosomes	SCC (36), Ctr (36)	Diagnostic SCC vs. Ctr	AUC = 0.842	
miR-181a-5p and miR-574-5p	Plasma EVs	Advanced NSCLC (245)	Prognostic NSCLC with nivolumab therapeutic	OS = 9 months, AUC = 0.76	[48]
miR-200b-3p, miR-3124-5p, and miR-92b-5p	Serum exosomes	SCLC (126), Ctr (50)	Diagnostic (SCLC vs. Ctr)	AUC = 0.93	[19]
miR-1290 miR-29c-3p	Plasma exosomes	NSCLC (30), SCLC (8), BLD (19)	Diagnostic LC vs. BLD	AUC = 0.934 AUC = 0.868	[49]
miR-1290 miR-29c-3p			Diagnostic early-stage LC vs. BLD	AUC = 0.947 AUC = 0.895	
miR-1290 and miR-29c-3p			Diagnostic NSCLC vs. SCLC	AUC = 0.860	
miR-4497	Serum exosomes	NSCLC (84), BLL (30), Ctr (47)	Diagnostic NSCLC vs. Ctrl	AUC = 0.855, sensitivity 76.6%, and specificity 83.3%	[51]
			Diagnostic NSCLC vs. BLL	AUC = 0.748, sensitivity 73.3%, and specificity 72.6%	
			Prognostic Low levels = shorter OS	*p* < 0.05	
miR-1260b	Plasma exosomes	NSCLC (48), Ctr (48)	Prognostic High levels = shorter OS	*p* = 0.029	[37]
miR-342-5p and miR-574-5p	Plasma exosomes	AD (56), Ctr (40)	Diagnostic early-stage AD vs. Ctrl	AUC = 0.813, sensitivity 80.0%, and specificity 73.2%	[50]
miR-5684 and miR-125b-5p	Serum exosomes	NSCLC (330), Ctr (332)	Diagnostic NSCLC vs. Ctrl	AUC = 0.793, sensitivity 82.7%, and specificity 62.1%	[52]
			Diagnostic early-stage NSCLC vs. Ctrl	AUC = 0.744, sensitivity 80.6%, and specificity 60.9%	
miR-7977	Serum exosomes	AD (62), Ctr (62)	Diagnostic AD vs. Ctrl	AUC = 0.787	[53]
miR-378	Serum exosomes	NSCLC (103), Ctr (60)	Diagnostic NSCLC vs. Ctrl	AUC = 0.842	[54]
			High levels = shorter OS	*p* < 0.001	

EVs—extracellular vesicles; LC—lung cancer; NSCLC—non-small-cell lung cancer; SCLC—small-cell lung cancer; AD—lung adenocarcinoma; SCC—lung squamous-cell carcinoma; AUC—the area under the curve; OS—overall survival; Ctr.—healthy controls; BLD—benign lung disease; BLL—benign lung lesion.

**Table 2:** Exosome-derived miRNAs from peripheral blood as potential predictive biomarkers for therapeutic response in lung cancer published over the last five years.

miRNAs	Sample	Cohort Size (n)	Application	Biomarker Performance Value	Ref
miR-184 and miR-3913-5p	Serum exosomes	NSCLC (67)	Biomarker for NSCLC patients resistant to osimertinib	AUC = 0.7059	[56]
			Biomarker for osimertinib resistance for patients with EGFR exon21 L858R mutation	AUC = 0.736 AUC = 0.759	
miR-199a-3p, miR-21–5p, and miR-28–5p	Plasma EVs	NSCLC (50): 22 responders and 28 non-responders.	Predictive biomarkers for anti-PD-1/PD-L1 treatment response	AUC = 0.925	[57]
miR-320d, miR-320c, miR-320b, and miR-125b-5p	Plasma exosomes	Advanced EGFR/ALK wild-type NSCLC (30)	Upregulated in non-responders after anti-PD-1/PD-L1 therapeutic (*p* < 0.05)	Not determined	[18]
miR-323-3p, miR-1468-3p, miR-5189-5p, and miR-6513-5p	Plasma exosomes	NSCLC (27)	Upregulated in osimertinib-resistant NSCLC patients (*p* < 0.0001)	Not determined	[58]

EVs—extracellular vesicles; NSCLC—non-small-cell lung cancer; AUC—the area under the curve; EGFR—epidermal growth factor receptor. PD—progressive disease; PR—partial response; PD-1—programmed cell death protein 1; PD-L1—programmed cell death protein 1.

**Table 3:** Exosome-derived miRNAs from pleural effusion and pleural lavage as potential lung cancer biomarkers.

miRNAs	Sample	Cohort Size (n)	Application	Biomarker Performance Value	Ref
miR-150-5p, miR- 3614-5p, miR-200b-3p, miR-182-5p, and miR-629-5p	Pleural effusion exosomes	AD (6), TB (6)	Diagnostic AD vs. TB	Not determined	[61]
miR-21	Pleural lavage EVs	AD (41)	Diagnostic High levels associated with positive cytology and pleural invasion	Not determined	[62]
miR-1-3p	Pleural lavage EVs	NSCLC (21), BPE patients (25)	Diagnostic NSCLC vs. BPE	AUC = 0.914	[63]
miR-144-5p				AUC = 0.925	
miR-150-5p				AUC = 0.939	

TB—pulmonary tuberculosis; AD—lung adenocarcinoma; EVs—extracellular vesicles; BPE—benign pleural effusion; NSCLC—non-small-cell lung cancer; AUC—area under the curve.

## References

[1] World Health Organization (WHO), International Agency for Research of Cancer (IARC) GLOBOCAN 2020, Section of Cancer Surveillance. http://globocan.iarc.fr.

[2] American Cancer Society Lung Cancer Survival Rates. https://www.cancer.org/cancer/types/lung-cancer/detection-diagnosis-staging/survival-rates.html.

[3] International Agency for Research on Cancer (iarc) WHO Cancer Today. http://gco.iarc.fr/today/home.

[4] Araghi M., Mannani R., Heidarnejad Maleki A., Hamidi A., Rostami S., Safa S.H., Faramarzi F., Khorasani S., Alimohammadi M., Tahmasebi S. (2023). Recent advances in non-small cell lung cancer targeted therapy; an update review. Cancer Cell Int..

[5] Miao D., Zhao J., Han Y., Zhou J., Li X., Zhang T., Li W., Xia Y. (2024). Management of locally advanced non-small cell lung cancer: State of the art and future directions. Cancer Commun..

[6] Wu L., Zhang Z., Bai M., Yan Y., Yu J., Xu Y. (2023). Radiation combined with immune checkpoint inhibitors for unresectable locally advanced non-small cell lung cancer: Synergistic mechanisms, current state, challenges, and orientations. Cell Commun. Signal.

[7] Li W., Liu J.B., Hou L.K., Yu F., Zhang J., Wu W., Tang X.M., Sun F., Lu H.M., Deng J. (2022). Liquid biopsy in lung cancer: Significance in diagnostics, prediction, and treatment monitoring. Mol. Cancer.

[8] Ren F., Fei Q., Qiu K., Zhang Y., Zhang H., Sun L. (2024). Liquid biopsy techniques and lung cancer: Diagnosis, monitoring and evaluation. J. Exp. Clin. Cancer Res..

[9] Siravegna G., Mussolin B., Venesio T., Marsoni S., Seoane J., Dive C., Papadopoulos N., Kopetz S., Corcoran R.B., Siu L.L. (2019). How liquid biopsies can change clinical practice in oncology. Ann. Oncol..

[10] Ignatiadis M., Sledge G.W., Jeffrey S.S. (2021). Liquid biopsy enters the clinic-implementation issues and future challenges. Nat. Rev. Clin. Oncol..

[11] Connal S., Cameron J.M., Sala A., Brennan P.M., Palmer D.S., Palmer J.D., Perlow H., Baker M.J. (2023). Liquid biopsies: The future of cancer early detection. J. Transl. Med..

[12] De Rubis G., Rajeev Krishnan S., Bebawy M. (2019). Liquid Biopsies in Cancer Diagnosis, Monitoring, and Prognosis. Trends Pharmacol. Sci..

[13] Yu W., Hurley J., Roberts D., Chakrabortty S.K., Enderle D., Noerholm M., Breakefield X.O., Skog J.K. (2021). Exosome-based liquid biopsies in cancer: Opportunities and challenges. Ann. Oncol..

[14] Li C., Zhou T., Chen J., Li R., Chen H., Luo S., Chen D., Cai C., Li W. (2022). The role of Exosomal miRNAs in cancer. J. Transl. Med..

[15] Ortiz-Quintero B. (2020). Extracellular MicroRNAs as Intercellular Mediators and Noninvasive Biomarkers of Cancer. Cancers.

[16] Xu D., Di K., Fan B., Wu J., Gu X., Sun Y., Khan A., Li P., Li Z. (2022). MicroRNAs in extracellular vesicles: Sorting mechanisms, diagnostic value, isolation, and detection technology. Front. Bioeng. Biotechnol..

[17] Jin X., Chen Y., Chen H., Fei S., Chen D., Cai X., Liu L., Lin B., Su H., Zhao L. (2017). Evaluation of Tumor-Derived Exosomal miRNA as Potential Diagnostic Biomarkers for Early-Stage Non-Small Cell Lung Cancer Using Next-Generation Sequencing. Clin. Cancer Res..

[18] Peng X.X., Yu R., Wu X., Wu S.Y., Pi C., Chen Z.H., Zhang X.C., Gao C.Y., Shao Y.W., Liu L. (2020). Correlation of plasma exosomal microRNAs with the efficacy of immunotherapy in EGFR/ALK wild-type advanced non-small cell lung cancer. J. Immunother. Cancer.

[19] Kim D.H., Park H., Choi Y.J., Im K., Lee C.W., Kim D.S., Pack C.G., Kim H.Y., Choi C.M., Lee J.C. (2023). Identification of exosomal microRNA panel as diagnostic and prognostic biomarker for small cell lung cancer. Biomark. Res..

[20] Tarasov V.V., Svistunov A.A., Chubarev V.N., Dostdar S.A., Sokolov A.V., Brzecka A., Sukocheva O., Neganova M.E., Klochkov S.G., Somasundaram S.G. (2021). Extracellular vesicles in cancer nanomedicine. Semin. Cancer Biol..

[21] Kalluri R., LeBleu V.S. (2020). The biology, function, and biomedical applications of exosomes. Science.

[22] Pathan M., Fonseka P., Chitti S.V., Kang T., Sanwlani R., Van Deun J., Hendrix A., Mathivanan S. (2019). Vesiclepedia 2019: A compendium of RNA, proteins, lipids and metabolites in extracellular vesicles. Nucleic Acids Res..

[23] Juan T., Furthauer M. (2018). Biogenesis and function of ESCRT-dependent extracellular vesicles. Semin. Cell Dev. Biol..

[24] Hessvik N.P., Llorente A. (2018). Current knowledge on exosome biogenesis and release. Cell. Mol. Life Sci..

[25] Mathieu M., Martin-Jaular L., Lavieu G., Thery C. (2019). Specificities of secretion and uptake of exosomes and other extracellular vesicles for cell-to-cell communication. Nat. Cell Biol..

[26] Shang R., Lee S., Senavirathne G., Lai E.C. (2023). microRNAs in action: Biogenesis, function and regulation. Nat. Rev. Genet..

[27] Bofill-De Ros X., Vang Orom U.A. (2024). Recent progress in miRNA biogenesis and decay. RNA Biol..

[28] Wilson R.C., Tambe A., Kidwell M.A., Noland C.L., Schneider C.P., Doudna J.A. (2015). Dicer-TRBP complex formation ensures accurate mammalian microRNA biogenesis. Mol. Cell.

[29] Wu K., He J., Pu W., Peng Y. (2018). The Role of Exportin-5 in MicroRNA Biogenesis and Cancer. Genom. Proteom. Bioinform..

[30] Schirle N.T., Sheu-Gruttadauria J., MacRae I.J. (2014). Structural basis for microRNA targeting. Science.

[31] Bartel D.P. (2009). MicroRNAs: Target recognition and regulatory functions. Cell.

[32] Kosaka N., Iguchi H., Hagiwara K., Yoshioka Y., Takeshita F., Ochiya T. (2013). Neutral sphingomyelinase 2 (nSMase2)-dependent exosomal transfer of angiogenic microRNAs regulate cancer cell metastasis. J. Biol. Chem..

[33] Koppers-Lalic D., Hackenberg M., Bijnsdorp I.V., van Eijndhoven M.A.J., Sadek P., Sie D., Zini N., Middeldorp J.M., Ylstra B., de Menezes R.X. (2014). Nontemplated nucleotide additions distinguish the small RNA composition in cells from exosomes. Cell Rep..

[34] Villarroya-Beltri C., Gutierrez-Vazquez C., Sanchez-Cabo F., Perez-Hernandez D., Vazquez J., Martin-Cofreces N., Martinez-Herrera D.J., Pascual-Montano A., Mittelbrunn M., Sanchez-Madrid F. (2013). Sumoylated hnRNPA2B1 controls the sorting of miRNAs into exosomes through binding to specific motifs. Nat. Commun..

[35] Martinez-Espinosa I., Serrato J.A., Ortiz-Quintero B. (2023). The Role of Exosome-Derived microRNA on Lung Cancer Metastasis Progression. Biomolecules.

[36] Martinez-Espinosa I., Serrato J.A., Ortiz-Quintero B. (2024). MicroRNAs in Lung Cancer Brain Metastasis. Int. J. Mol. Sci..

[37] Kim D.H., Park H., Choi Y.J., Kang M.H., Kim T.K., Pack C.G., Choi C.M., Lee J.C., Rho J.K. (2021). Exosomal miR-1260b derived from non-small cell lung cancer promotes tumor metastasis through the inhibition of HIPK2. Cell Death Dis..

[38] Wei L., Wang G., Yang C., Zhang Y., Chen Y., Zhong C., Li Q. (2021). MicroRNA-550a-3-5p controls the brain metastasis of lung cancer by directly targeting YAP1. Cancer Cell Int..

[39] Liu J., Cao L., Li Y., Deng P., Pan P., Hu C., Yang H. (2022). Pirfenidone promotes the levels of exosomal miR-200 to down-regulate ZEB1 and represses the epithelial-mesenchymal transition of non-small cell lung cancer cells. Hum. Cell.

[40] Morrissey S.M., Zhang F., Ding C., Montoya-Durango D.E., Hu X., Yang C., Wang Z., Yuan F., Fox M., Zhang H.G. (2021). Tumor-derived exosomes drive immunosuppressive macrophages in a pre-metastatic niche through glycolytic dominant metabolic reprogramming. Cell Metab..

[41] Yu F., Liang M., Huang Y., Wu W., Zheng B., Chen C. (2021). Hypoxic tumor-derived exosomal miR-31-5p promotes lung adenocarcinoma metastasis by negatively regulating SATB2-reversed EMT and activating MEK/ERK signaling. J. Exp. Clin. Cancer Res..

[42] He S., Li Z., Yu Y., Zeng Q., Cheng Y., Ji W., Xia W., Lu S. (2019). Exosomal miR-499a-5p promotes cell proliferation, migration and EMT via mTOR signaling pathway in lung adenocarcinoma. Exp. Cell Res..

[43] Liu Y., Su C.Y., Yan Y.Y., Wang J., Li J.J., Fu J.J., Wang Y.Q., Zhang J.Y. (2022). Exosomes of A549 Cells Induced Migration, Invasion, and EMT of BEAS-2B Cells Related to let-7c-5p and miR-181b-5p. Front. Endocrinol..

[44] Siravegna G., Marsoni S., Siena S., Bardelli A. (2017). Integrating liquid biopsies into the management of cancer. Nat. Rev. Clin. Oncol..

[45] Yang D., Zhang W., Zhang H., Zhang F., Chen L., Ma L., Larcher L.M., Chen S., Liu N., Zhao Q. (2020). Progress, opportunity, and perspective on exosome isolation-efforts for efficient exosome-based theranostics. Theranostics.

[46] Chen J., Li P., Zhang T., Xu Z., Huang X., Wang R., Du L. (2021). Review on Strategies and Technologies for Exosome Isolation and Purification. Front. Bioeng. Biotechnol..

[47] Hassanin A.A.I., Ramos K.S. (2024). Circulating Exosomal miRNA Profiles in Non-Small Cell Lung Cancers. Cells.

[48] Genova C., Marconi S., Chiorino G., Guana F., Ostano P., Santamaria S., Rossi G., Vanni I., Longo L., Tagliamento M. (2024). Extracellular vesicles miR-574-5p and miR-181a-5p as prognostic markers in NSCLC patients treated with nivolumab. Clin. Exp. Med..

[49] Zhang Q., Zheng K., Gao Y., Zhao S., Zhao Y., Li W., Nan Y., Li Z., Liu W., Wang X. (2023). Plasma exosomal miR-1290 and miR-29c-3p as diagnostic biomarkers for lung cancer. Heliyon.

[50] Han Z., Li Y., Zhang J., Guo C., Li Q., Zhang X., Lan Y., Gu W., Xing Z., Liang L. (2020). Tumor-derived circulating exosomal miR-342-5p and miR-574-5p as promising diagnostic biomarkers for early-stage Lung Adenocarcinoma. Int. J. Med. Sci..

[51] Zheng B., Peng M., Gong J., Li C., Cheng H., Li Y., Tang Y. (2023). Circulating exosomal microRNA-4497 as a potential biomarker for metastasis and prognosis in non-small-cell lung cancer. Exp. Biol. Med..

[52] Zhang Z., Tang Y., Song X., Xie L., Zhao S., Song X. (2020). Tumor-Derived Exosomal miRNAs as Diagnostic Biomarkers in Non-Small Cell Lung Cancer. Front. Oncol..

[53] Chen L., Cao P., Huang C., Wu Q., Chen S., Chen F. (2020). Serum exosomal miR-7977 as a novel biomarker for lung adenocarcinoma. J. Cell. Biochem..

[54] Zhang Y., Xu H. (2020). Serum exosomal miR-378 upregulation is associated with poor prognosis in non-small-cell lung cancer patients. J. Clin. Lab. Anal..

[55] Wang N., Guo W., Song X., Liu L., Niu L., Song X., Xie L. (2020). Tumor-associated exosomal miRNA biomarkers to differentiate metastatic vs. nonmetastatic non-small cell lung cancer. Clin. Chem. Lab. Med..

[56] Li X., Chen C., Wang Z., Liu J., Sun W., Shen K., Lv Y., Zhu S., Zhan P., Lv T. (2021). Elevated exosome-derived miRNAs predict osimertinib resistance in non-small cell lung cancer. Cancer Cell Int..

[57] Shukuya T., Ghai V., Amann J.M., Okimoto T., Shilo K., Kim T.K., Wang K., Carbone D.P. (2020). Circulating MicroRNAs and Extracellular Vesicle-Containing MicroRNAs as Response Biomarkers of Anti-programmed Cell Death Protein 1 or Programmed Death-Ligand 1 Therapy in NSCLC. J. Thorac. Oncol..

[58] Janpipatkul K., Trachu N., Watcharenwong P., Panvongsa W., Worakitchanon W., Metheetrairut C., Oranratnachai S., Reungwetwattana T., Chairoungdua A. (2021). Exosomal microRNAs as potential biomarkers for osimertinib resistance of non-small cell lung cancer patients. Cancer Biomark..

[59] Kim J.E., Eom J.S., Kim W.Y., Jo E.J., Mok J., Lee K., Kim K.U., Park H.K., Lee M.K., Kim M.H. (2018). Diagnostic value of microRNAs derived from exosomes in bronchoalveolar lavage fluid of early-stage lung adenocarcinoma: A pilot study. Thorac. Cancer.

[60] Rehbein G., Schmidt B., Fleischhacker M. (2015). Extracellular microRNAs in bronchoalveolar lavage samples from patients with lung diseases as predictors for lung cancer. Clin. Chim. Acta.

[61] Zhang X., Bao L., Yu G., Wang H. (2022). Exosomal miRNA-profiling of pleural effusion in lung adenocarcinoma and tuberculosis. Front. Surg..

[62] Watabe S., Kikuchi Y., Morita S., Komura D., Numakura S., Kumagai-Togashi A., Watanabe M., Matsutani N., Kawamura M., Yasuda M. (2020). Clinicopathological significance of microRNA-21 in extracellular vesicles of pleural lavage fluid of lung adenocarcinoma and its functions inducing the mesothelial to mesenchymal transition. Cancer Med..

[63] Roman-Canal B., Moiola C.P., Gatius S., Bonnin S., Ruiz-Miro M., Gonzalez E., Ojanguren A., Recuero J.L., Gil-Moreno A., Falcon-Perez J.M. (2019). EV-associated miRNAs from pleural lavage as potential diagnostic biomarkers in lung cancer. Sci. Rep..

[64] Welsh J.A., Goberdhan D.C.I., O’Driscoll L., Buzas E.I., Blenkiron C., Bussolati B., Cai H., Di Vizio D., Driedonks T.A.P., Erdbrugger U. (2024). Minimal information for studies of extracellular vesicles (MISEV2023): From basic to advanced approaches. J. Extracell. Vesicles.

